# Association of the Korean-specific food-based index of dietary inflammatory potential with the risk of mild cognitive impairment in Korean older adults

**DOI:** 10.4178/epih.e2024067

**Published:** 2024-07-25

**Authors:** Se Yeon Hwang, Chong-Su Kim, Mi Kyung Kim, Yoonkyoung Yang, Yoon Jung Yang

**Affiliations:** 1 Department of Clinical Nutrition, Dongduk Women’s University, Seoul, Korea; 2 Department of Food and Nutrition, Dongduk Women’s University, Seoul, Korea; 3 Department of Preventive Medicine, Hanyang University College of Medicine, Seoul, Korea; 4 Department of Food and Nutrition, Ansan University, Ansan, Korea

**Keywords:** Mild cognitive impairment, Anti-inflammatories, Elderly

## Abstract

**OBJECTIVES:**

This study aimed to examine the association between the food-based index of dietary inflammatory potential (FBDI) and the risk of mild cognitive impairment (MCI) in Korean older adults.

**METHODS:**

The subjects were 798 Korean adults aged 60 years and older. The FBDI was calculated based on the intake of 7 anti-inflammatory and 3 inflammatory food groups. Cognitive function was assessed using the Korean version of the Mini-Mental State Examination. A general linear model and multiple logistic regression were applied to assess the association between FBDI and the risk of MCI.

**RESULTS:**

As the FBDI increased, the intake of white rice, cookies/candies, and sweetened drinks tended to increase, but the intake of niacin, β-carotene, calcium, and potassium tended to decrease (p for trend<0.05). The highest FBDI group had a higher MCI risk (odds ratio [OR], 1.60; 95% confidence interval [CI], 1.01 to 2.52) than the lowest FBDI group, adjusted for gender, age, and education level; and this trend was significant in a fully adjusted model (p for trend=0.039). No significant associations were found in men after adjusting for confounding factors. Among women, MCI risk increased as the FBDI increased (p for trend=0.007); and the highest FBDI group had a higher MCI risk (OR, 2.22; 95% CI, 1.04 to 4.74) than the lowest FBDI group in a fully adjusted model.

**CONCLUSIONS:**

These results suggest that the appropriate intake of anti-inflammatory foods and nutrients may be associated with a reduced risk of MCI among older adults.

## GRAPHICAL ABSTRACT

[Fig f1-epih-46-e2024067]

## Key Message

Our study investigated the association between the Korean-specific food-based index of dietary inflammatory potential (FBDI) and risk of mild cognitive impairment (MCI) in adults aged 60 and older. The FBDI was calculated based on the intake of 7 anti-inflammatory and 3 inflammatory food groups. The result showed that older adults with a higher FBDI tended to have a higher risk of MCI. These findings suggest that the appropriate intake of anti-inflammatory foods and nutrients may be associated with a reduced risk of MCI among older adults.

## INTRODUCTION

The global population of older adults is experiencing a significant increase, and projections indicate that by 2050, the number of people aged 65 and older will have doubled compared to 2021 [1]. The rate of growth in the older population is expected to be fastest in Korea [1], where the percentage of individuals aged 65 years and older is anticipated to rise from 17.5% in 2022 to 46.4% by 2070 [2]. Cognitive decline is increasingly recognized as a major health concern among older adults [3]. Mild cognitive impairment (MCI) represents a transitional stage between normal aging and dementia [4]. Since MCI is a high-risk precursor to dementia, with an annual progression rate of 12% [5], proactive intervention during the predementia stage is essential [6].

Cognitive impairment has been closely linked to inflammation, as shown by elevated levels of C-reactive protein (CRP), interleukin (IL)-6, and tumor necrosis factor (TNF)-α in affected individuals [7-9]. Furthermore, inflammation has been identified as a potential contributor to cognitive impairment [10] and is closely associated with dietary factors [11]. Shivappa et al. [12] created the Dietary Inflammatory Index (DII), which correlates 45 food parameters with 6 inflammation markers (IL-1β, IL-6, TNF-α, CRP, IL-4, IL-10) based on a comprehensive literature review. Previous research has demonstrated that higher DII scores are linked to an increased risk of cognitive impairment [13-15]. However, the DII, primarily developed in the United States and Europe, focuses on inflammation markers influenced by nutrients and may not accurately reflect the dietary habits of Koreans. This discrepancy arises because the commonly consumed foods vary significantly between cultures [12]. To address this issue, Korean researchers developed the food-based index of dietary inflammatory potential (FBDI), which takes into account the dietary patterns of Koreans and explores the relationship between diet and inflammation potential [16]. In Korea, the traditional carbohydrate-based dietary pattern includes rice as a staple, along with soup/stew and side dishes, and has been a longstanding practice [17]. Consequently, there is a notable difference in the proportion of energy derived from the 3 major nutrients when compared to Western diets [18]. Since the FBDI incorporates the top 30 most frequently consumed foods in Korea [19], it is deemed more appropriate than the DII for assessing diet-related inflammation among Koreans.

Although previous studies have reported a negative association between diet-related inflammation and cognitive function in older adults [20], research exploring the relationship between inflammatory markers specific to the Korean diet and cognitive function remains scarce. Therefore, this study aimed to examine the association between the FBDI and the risk of MCI in Korean older adults, and to provide evidence supporting the importance of nutritional management in the elderly population to prevent cognitive dysfunction.

## MATERIALS AND METHODS

### Data source and study population

This cross-sectional study analyzed data collected from the Yangpyeong cohort study between July 2009 and August 2010. Out of 1,638 participants, 808 individuals aged 60 years and older who participated in the Korean version of the Mini-Mental State Examination (MMSE-KC) were selected for analysis. We further excluded participants who reported energy consumption of less than 500 kcal/day (n=2) and those with incomplete data on health questionnaires and examinations (n=8). Therefore, the final analysis included a total of 798 participants, comprising 334 men and 464 women.

### General characteristics and anthropometric data

Trained interviewers conducted in-person interviews using a standardized protocol to gather information on various factors including education level, living status, social activity, drinking status, smoking habits, regular exercise, and supplement use. Additionally, they collected data on medical history, which included previously diagnosed conditions such as hypertension, hyperlipidemia, diabetes mellitus, cardiovascular disease, and stroke—conditions known to influence cognitive function. Participants were asked, “Have you ever been diagnosed with any diseases by a doctor in a clinic or hospital?”. Examiners also measured height, weight, and waist circumference following standard procedures. Body mass index (BMI) was calculated by dividing the weight in kilograms by the square of the height in meters (kg/m^2^). Education level was categorized into 4 groups: “uneducated,” “elementary school,” “middle and high school,” and “college or higher”. Living status was classified as either “alone” or “with spouse or family”. Social activity was simply classified as “yes” or “no”. Drinking status was categorized into “non-drinker,” “former drinker,” and “current drinker”. For smoking habits, participants were asked, “Have you smoked a total of 20 packs (400 cigarettes) or more in your lifetime?”. Responses were categorized as “non-smoker,” “former smoker,” or “current smoker”. Regular exercise was determined by the question, “Do you regularly engage in exercise that makes you sweat?” with responses of “yes” or “no”. Supplement use was classified based on the intake of 1 or more of the following: multivitamins, vitamins A, C, E, B, β-carotene, calcium, iron, zinc, folic acid, selenium, vitamin D, copper, magnesium, squalene, omega-3 fatty acids, other supplements, and health supplements.

### Dietary assessment

The quantitative food frequency questionnaire (FFQ), which has been validated for both validity and reliability, was used to assess nutritional intake [21]. This FFQ captured the average frequency and quantity of consumption over the past year, covering 106 food items. Consumption frequencies were categorized into 9 groups: almost never, once/mo, 2-3 times/mo, 1-2 times/wk, 3-4 times/wk, 5-6 times/wk, once/day, 2 times/day, and 3 times/day. Portion sizes were classified into 3 categories: 0.5 servings, 1.0 serving, and 1.5 servings. To determine the daily serving size, we converted the frequency category selected for each food item into a daily consumption rate. The daily serving size, along with the energy and nutrients consumed, was calculated by multiplying the daily consumption frequency by the portion size selected. Following the methodology of Yoon et al. [22], we reclassified 18 food groups to analyze their relation to inflammation. These groups included white rice, rice with other cereals, noodles/rice cakes, breads, cereals, cookies/candies, legumes/nut seeds, potatoes, kimchi, vegetables, red/processed meats, poultry/eggs, blue-backed fish, fish/seafood, dairy products, sweetened drinks, other drinks, and fruits. Nutrient intake was adjusted for total energy using the residual method [23].

### Food-based index of dietary inflammatory potential

A dietary inflammation assessment tool, developed by Na et al. [16], tailored to Korean dietary habits, was utilized to categorize FBDI groups. The FBDI calculation involved using regression coefficients derived from correlation and multiple regression analyses. These analyses examined the relationship between the consumption of 7 anti-inflammatory food groups—bread/wheat flour, nuts/seeds, legumes, eggs, green vegetables, citrus fruits, red berries—and 3 inflammatory food groups—white rice, beef, mixed coffee/sweetened drinks—and their impact on high-sensitivity C-reactive protein (hs-CRP) levels [16]. The specific food items comprising each group have been detailed previously [22]. The formula used to calculate the FBDI is as follows:

The FBDI is calculated using the following formula: (White rice applied value [0.024]× White rice intake [g])+(Bread/wheat flour applied value [-0.035]× Bread/wheat flour intake [g])+(Nuts/seeds applied value [-0.089]× Nuts/seeds intake [g])+(Legumes applied value [-0.027]× Legumes intake [g])+(Eggs applied value [-0.063]×Eggs intake [g])+(Green vegetables applied value [-0.034]× Green vegetables intake [g])+(Beef applied value [0.040]×Beef intake [g])+(Citrus fruits applied value [-0.032]× Citrus fruits intake [g])+(Red berries applied value [-0.015]× Red berries intake [g])+(Mixed coffee/sweetened drinks applied value [0.029]×Mixed coffee/sweetened drinks intake [g]).

### Cognitive function assessment

The Mini-Mental State Examination (MMSE) is a widely used assessment tool, validated for its reliability and effectiveness in evaluating cognitive abilities [24]. Cognitive function was assessed using the MMSE-KC [25]. The MMSE-KC includes various components: time and place orientation (10 items, 10 points), concentration (1 item, 5 points), memory registration (1 item, 3 points), memory recall (1 item, 3 points), naming and repetition (2 items, 3 points), 3-stage command (1 item, 3 points), interlocking pentagon copying (1 item, 1 point), and discernment (2 items, 2 points). The maximum possible score on the MMSE-KC is 30 points, with higher scores indicating better cognitive abilities. The criteria for determining the presence of MCI using MMSE-KC scores take into account age, gender, and educational level. Subjects with MMSE-KC scores equal to or greater than -1.5 standard deviations (SD) from the mean of the general population were classified as having normal cognitive function. Conversely, those with MMSE-KC scores less than -1.5 SD from the mean were identified as having MCI [26].

### Statistical analysis

Frequency and percentage were used to represent categorical variables, while continuous variables were expressed as mean and SD. The chi-square test was employed to analyze categorical variables based on the subjects’ FBDI quartiles, and analysis of variance was used for continuous variables. When more than 20% of the cells had an expected count of less than 5, the Fisher exact test was utilized. The Tukey multiple comparison test was conducted for the post-hoc analysis. A general linear model was applied to determine the p-for-trend across the FBDI quartiles of subjects after adjusting for covariates. Multiple logistic regression analysis was used to evaluate the association between FBDI and mild cognitive impairment. Adjusted odds ratios (ORs) along with 95% confidence intervals (CIs) were presented, accounting for variables such as gender, age, education level, exercise, alcohol drinking, and use of supplements. The variables ‘smoking habits’ and ‘medical history’ were not included as covariates due to the use of only fully observed data, stemming from a limited number of respondents. In contrast, the ‘use of supplements’ variable, which had relatively fewer missing values, was incorporated into the model. All statistical analyses were performed using the SAS version 9.4 (SAS Institute Inc., Cary, NC, USA). A p-value of less than 0.05 was considered statistically significant.

### Ethics statement

All procedures were conducted according to the Institutional Review Board of Hanyang University (HYI-12-038-revision2). Written informed consent was obtained from all participants.

## RESULTS

### General characteristics

Table 1 presents the general characteristics of the study subjects, categorized by quartile of FBDI. The group with the highest FBDI had a significantly higher proportion of men and a lower proportion of women compared to the group with the lowest FBDI (p<0.001). Additionally, the highest FBDI group was, on average, significantly older than the lowest FBDI group (p<0.001). However, there were no significant differences in height and weight between these 2 groups. There were also no significant differences in BMI, waist circumference, living status, social activity, and smoking habits between the groups. The highest FBDI group, compared to the lowest, contained a significantly higher proportion of uneducated individuals (p<0.001) and current drinkers (p=0.024). Conversely, this group had a lower proportion of individuals who engage in exercise and take supplements (p<0.001). Significant differences were observed in the prevalence of hypertension (p=0.021) and diabetes mellitus (p=0.017) among the FBDI groups. However, there were no significant differences in the prevalence of hyperlipidemia, cardiovascular disease, and stroke.

### Food group intakes according to food-based index of dietary inflammatory potential

Table 2 displays the intake of various food groups across different FBDI groups. As the FBDI increased, there was a tendency for the consumption of white rice, cookies/candies, and sweetened drinks to increase. Conversely, the intake of rice with other cereals, breads, legumes/nut seeds, potatoes, kimchi, vegetables, fish/seafood, and fruits generally decreased (p for trend < 0.05). However, no significant trends were observed in the consumption of noodles/rice cakes, cereals, red/processed meats, poultry/eggs, blue-backed fish, dairy products, and other drinks.

### Nutrient intakes according to food-based index of dietary inflammatory potential

The nutritional characteristics of the FBDI groups are detailed in Table 3, which compares calorie and nutrient intakes across each group. As the FBDI increased, there was a general decrease in the intake of protein, all fat, cholesterol, calcium, phosphorus, iron, potassium, zinc, vitamin A, retinol, β-carotene, vitamin B1, vitamin B2, niacin, vitamin B6, folate, vitamin C, vitamin E, and fiber, with these trends being statistically significant (p for trend < 0.05). However, the intakes of energy, carbohydrate, and sodium did not show significant trends.

### Korean version of the Mini-Mental State Examination scores according to food-based index of dietary inflammatory potential

To investigate the relationship between dietary intake and the risk of MCI in older adults, we assessed the association between the FBDI and MMSE-KC scores using the general linear model. Table 4 displays the average MMSE-KC scores of the study subjects categorized by FBDI. Overall, there was a tendency for the average MMSE-KC scores to decrease as FBDI increased, after adjusting for factors such as gender, age, and education level (p for trend=0.013). However, this trend was not significant when fully adjusted for confounding factors in model 2. We also explored gender differences in the relationship between MMSE-KC scores and FBDI. Among men, the average MMSE-KC scores decreased with an increase in FBDI after adjustments for age and education level (p for trend=0.013). However, this association was not significant upon full adjustment for confounding factors in model 2. Among women, no significant associations were found when adjusted for confounding factors in both model 1 and model 2.

### Adjusted odds ratios and 95% confidence intervals of mild cognitive impairment according to food-based index of dietary inflammatory potential

Adjusted ORs and 95% CIs of MCI by FBDI are presented in Table 5. Among all participants, those in the highest FBDI group exhibited a higher risk of MCI compared to those in the lowest group, after adjustments for gender, age, and education level (Q4 vs. Q1, OR, 1.60; 95% CI, 1.01 to 2.52). Additionally, the risk of MCI appeared to increase with higher FBDI when fully adjusted for confounding factors in model 2 (p for trend=0.039). We also investigated potential gender differences. No significant associations were found among men in either model 1 or model 2 after adjusting for confounding factors. However, among women, the risk of MCI tended to rise with increasing FBDI after adjustments for age and education level (p for trend=0.047). Moreover, women in the highest FBDI group had a significantly higher risk of MCI than those in the lowest group (Q4 vs. Q1, OR, 2.22; 95% CI, 1.04 to 4.74) after comprehensive adjustment for confounding variables in model 2.

## DISCUSSION

This study aimed to investigate the association between FBDI and the risk of MCI among older adults using data from the Yangpyeong cohort study. A tendency was observed for the risk of MCI to increase as the FBDI group shifted from low to high, with this trend being more prominent in women.

In the present study, we utilized the FBDI, which incorporates the most commonly consumed food groups among Koreans. We found that participants with higher FBDI scores consumed more white rice, cookies/candies, and sweetened drinks, but less rice with other cereals, legumes/nut seeds, vegetables, fish/seafood, and fruits compared to those with lower FBDI scores. These findings are consistent with previous research, confirming the FBDI’s effectiveness in indicating inflammatory markers [16]. Indices for diet-related inflammatory potential have been developed in various countries, establishing a link between diet and inflammatory biomarkers in the blood. A Finnish study involving 756 adults showed that an increase of 50 g/day in whole grain intake was associated with a 0.12 mg/L reduction in hs-CRP, whereas an equivalent increase in refined grain intake led to a 0.23 mg/L increase in hs-CRP [27]. Tamez et al. [28] observed a 50% higher serum CRP concentration in 825 Mexican women who consumed the most soda. Previous studies have consistently demonstrated that increased vegetable intake is associated with lower levels of hs-CRP, TNF-α, and IL-6 [29-31]. Additionally, a study on Iranian adults found that consuming a cup of cooked soybeans 3 times a week resulted in a significant reduction in serum CRP levels after 8 weeks [32]. Collectively, these findings suggest that white rice and sweetened beverages are positively correlated with inflammatory markers, whereas fruits, vegetables, legumes, and whole grains are inversely associated with these markers. Analyzing nutritional patterns in relation to the inflammation assessment index revealed a negative association between the total daily intake of niacin, β-carotene, calcium, and potassium with FBDI. Niacin is essential for nerve myelination, brain calcium signaling, and acts as an antioxidant [33-35]. Studies have shown that consuming niacin-rich cereals and legumes reduces inflammation markers [36,37], which in turn affects brain function [38]. Nicotinamide, a form of niacin, significantly reduced IL-6 and TNF-α levels in a study involving human whole blood co-cultured with endotoxin in vitro [39]. Additionally, serum β-carotene levels have been reported to inversely correlate with inflammatory markers [40], and diets rich in vegetables and fruits, which are sources of β-carotene, help reduce levels of these markers [41]. In an animal study, rats administered a 10 mg/kg dose of β-carotene exhibited significantly reduced serum IL-6 and TNF-α levels, demonstrating its anti-inflammatory effects [42]. Other studies have suggested that dietary calcium and potassium may play a role in regulating oxidative and inflammatory stress [43,44]. Foods high in potassium, such as legumes, whole grains, and vegetables, contain anti-inflammatory and antioxidant compounds [45]. In another animal study, the gene expression of TNF-α and IL-6 was significantly suppressed in the high calcium group compared to the basal calcium group [43]. The expression of IL-6 was reduced by approximately 58% in the cortex of the group that consumed 2% potassium chloride, while strong expression was observed in the control group [44]. Together with previous research, our findings suggest that adequate intake of niacin, β-carotene, calcium, and potassium may be associated with anti-inflammatory effects, possibly by suppressing pro-inflammatory cytokines such as TNF-α and IL-6. Therefore, these results suggest that maintaining proper nutrient intake with anti-inflammatory properties may have a supportive impact on cognitive health.

While the exact cause of Alzheimer’s disease (AD) remains unknown [46], chronic inflammation is linked to the formation of amyloid-β (Aβ) peptide plaques [47]. Aβ is pivotal in promoting neurodegeneration in AD and triggering the inflammatory response that leads to synaptic alterations [47]. A characteristic of aging-related cognitive decline, mediated by neuroinflammation, is the alteration of the blood-brain barrier. This alteration permits peripheral inflammatory cells to infiltrate the brain [46]. Our study indicated a trend toward an increased risk of MCI with higher values of the FBDI.

The present study revealed that the link between FBDI and the risk of MCI was more pronounced in women than in men. Research involving Spanish older adults over the age of 65 found that women exhibited higher levels of depression compared to men [48]. It is commonly hypothesized that current depression, associated with reduced hippocampal size, leads to episodic memory loss and subsequently cognitive deficits in adults [49]. Additionally, a study investigating the effects of changes in hippocampal volume on the progression or likelihood of AD and MCI showed that an increase in hippocampal volume decreased the odds of developing AD, with a greater increase observed in women than in men [50]. Further follow-up studies are needed to clarify the relationship between FBDI and MCI risk according to gender.

This study has several limitations. First, it only includes older individuals from a specific area, Yangpyeong in Gyeonggi Province, which may not accurately represent the entire elderly population of Korea. However, the study does have the advantage of reflecting the characteristics of a rural population, but future research should include a more diverse range of regions. Second, as it is a cross-sectional study using data from the Yangpyeong cohort, it is not possible to establish a causal relationship between FBDI and cognitive function. Third, hs-CRP was the sole marker of inflammation used in the FBDI, which is less reliable compared to the 6 inflammation markers (IL-1β, IL-4, IL-6, IL-10, TNF-α, hs-CRP) used in the DII. Therefore, further studies are needed to develop a Korean dietary inflammation index that incorporates various inflammation markers. Fourth, despite our efforts to control for confounding variables, there may still have been residual confounding factors, such as unknown risk or protective elements, that could have impacted cognitive function.

Despite these limitations, this study identified an association between FBDI and the risk of mild cognitive impairment among older adults in Korea. FBDI, a dietary inflammation index based on food groups rather than nutrients, is particularly meaningful as it was developed in accordance with Korean dietary habits, unlike other inflammation indices based on food groups from the United States or Europe.

In conclusion, to maintain cognitive health in older individuals, it is necessary to ensure adequate consumption of anti-inflammatory foods such as whole grains, legumes, vegetables, and fruits. Given that cognitive impairment is a significant predictor of dementia, implementing anti-inflammatory dietary guidelines for older adults in Korea could help mitigate the risk of cognitive decline.

## Figures and Tables

**Figure f1-epih-46-e2024067:**
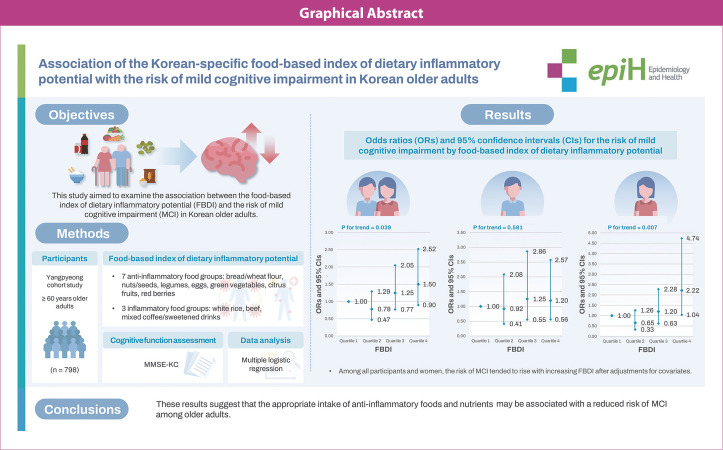


**Table 1.** General characteristics of study participants by quartile of food-based index of dietary inflammatory potential (FBDI)

Characteristics	FBDI				p-value^1^
	Quartile 1 (n=199)	Quartile 2 (n=200)	Quartile 3 (n=200)	Quartile 4 (n=199)	
FBDI (score)	-6.95±2.66	-2.89±0.65	-0.93±0.56	11.48±5.67	<0.001
Gender					<0.001
Men	80 (40.2)	68 (34.0)	70 (35.0)	116 (58.3)	
Women	119 (59.8)	132 (66.0)	130 (65.0)	83 (41.7)	
Age (yr)^2^	66.36±4.86^a^	67.69±5.19^a,b^	68.93±5.06^b^	68.34±6.04^b^	<0.001
Height (cm)^2^	157.34±8.47^b,c^	155.40±8.29^a,b^	154.95±8.30^a^	157.82±8.62^c^	0.001
Weight (kg)^2^	61.28±9.42^c^	58.44±9.55^a^	58.58±8.70^a,b^	60.42±9.21^a,c^	0.003
Body mass index (kg/m^2^)	24.74±3.20	24.14±3.06	24.41±3.28	24.24±3.10	0.253
Waist circumference (cm)	85.16±9.06	84.23±9.13	84.66±8.33	85.35±8.17	0.565
Education					<0.001
Uneducated	41 (20.6)	76 (38.0)	85 (42.5)	85 (42.7)	
Elementary school	76 (38.2)	69 (34.5)	82 (41.0)	60 (30.2)	
Middle and high school	63 (31.7)	46 (23.0)	28 (14.0)	48 (24.1)	
College or higher	19 (9.6)	9 (4.5)	5 (2.5)	6 (3.0)	
Living status					0.511
Alone	31 (15.6)	31 (15.5)	41 (20.5)	34 (17.1)	
With spouse or family	168 (84.4)	169 (84.5)	159 (79.5)	165 (82.9)	
Social activity (yes)	152 (76.4)	164 (82.0)	146 (73.0)	141 (70.9)	0.054
Drinking status					0.024
Non-drinker	112 (56.3)	115 (57.5)	109 (54.5)	82 (41.2)	
Former drinker	14 (7.0)	16 (8.0)	18 (9.0)	18 (9.1)	
Current drinker	73 (36.7)	69 (34.5)	73 (36.5)	99 (49.8)	
Smoking^3^					0.145
Non-smoker	26 (68.4)	40 (83.3)	35 (68.6)	25 (59.5)	
Former smoker	6 (15.8)	6 (12.5)	12 (23.5)	9 (21.4)	
Current smoker	6 (15.8)	2 (4.2)	4 (7.8)	8 (19.1)	
Exercise (yes)	94 (47.2)	65 (32.5)	43 (21.5)	30 (15.1)	<0.001
Use of supplements (yes)^4^	74 (40.9)	47 (25.7)	50 (28.6)	28 (17.5)	<0.001
Hypertension (yes)^5^	68 (64.2)	70 (60.3)	75 (62.5)	57 (46.3)	0.021
Hyperlipidemia (yes)^5^	6 (5.7)	6 (5.2)	6 (5.0)	5 (4.1)	0.954
Diabetes mellitus (yes)^5^	24 (22.6)	28 (24.1)	30 (25.0)	13 (10.6)	0.017
Cardiovascular disease (yes)^5^	13 (12.3)	12 (10.3)	18 (15.0)	11 (8.9)	0.490
Stroke (yes)^5^	7 (6.6)	4 (3.5)	2 (1.7)	8 (6.5)	0.171

Values are presented as number (%) or mean±standard deviation.

1 Analysis of variance for continuous variables and the chi-square test or Fisher exact test for categorical variables.

2 Different superscript letters (^a, b, c^) indicate that mean values were significantly different within a column, based on Tukey multiple comparison test.

3 Smoking (n=179).

4 Use of supplements (n=699).

5 Hypertension, hyperlipidemia, diabetes mellitus, cardiovascular disease, and stroke (n=465).

**Table 2.** Food group intakes of study participants by quartile of food-based index of dietary inflammatory potential (FBDI)

Food groups (servings/day)	FBDI				p for trend^1^
	Quartile 1 (n=199)	Quartile 2 (n=200)	Quartile 3 (n=200)	Quartile 4 (n=199)	
White rice	0.00±0.07	0.00±0.07	0.02±0.07	2.55±0.07	<0.001
Rice with other cereals	2.68±0.10	2.80±0.10	2.67±0.10	0.06±0.10	<0.001
Noodles/Rice cakes	0.31±0.03	0.25±0.03	0.20±0.03	0.27±0.03	0.944
Breads	0.19±0.02	0.08±0.02	0.07±0.02	0.08±0.02	0.004
Cereals	0.09±0.03	0.07±0.03	0.04±0.03	0.06±0.03	0.662
Cookies/Candies	1.75±0.16	1.63±0.16	1.77±0.16	2.03±0.16	0.010
Legumes/Nut seeds	1.96±0.12	1.11±0.12	0.70±0.12	1.03±0.13	<0.001
Potatoes	0.57±0.06	0.31±0.06	0.17±0.06	0.26±0.06	<0.001
Kimchi	3.17±0.23	2.69±0.23	2.38±0.24	2.39±0.24	0.004
Vegetables	3.86±0.22	2.19±0.22	1.48±0.22	2.11±0.23	<0.001
Red/Processed meats	0.30±0.03	0.23±0.04	0.20±0.04	0.28±0.04	0.523
Poultry/Eggs	0.05±0.01	0.03±0.01	0.02±0.01	0.04±0.01	0.770
Blue-backed fish	0.21±0.02	0.14±0.02	0.13±0.02	0.15±0.02	0.108
Fish/Seafood	2.15±0.13	1.34±0.13	1.05±0.13	1.29±0.14	<0.001
Dairy products	2.39±0.18	1.93±0.19	2.04±0.19	2.27±0.19	0.563
Sweetened drinks	1.83±0.16	1.72±0.16	1.96±0.16	2.16±0.17	0.006
Other drinks	0.70±0.09	0.47±0.09	0.39±0.09	0.47±0.10	0.104
Fruits	2.28±0.10	1.42±0.11	0.79±0.11	1.19±0.11	<0.001

Values are presented as least squares mean±standard deviation.

1 By general linear model after adjusting for gender, age, education, exercise, and alcohol drinking.

**Table 3.** Dietary nutrient intakes of study participants by quartile of FBDI

Nutrients	FBDI				p for trend^1^
	Quartile 1 (n=199)	Quartile 2 (n=200)	Quartile 3 (n=200)	Quartile 4 (n=199)	
Energy (kcal/day)	1,368.48±11.34	1,398.32±11.60	1,370.50±11.76	1,360.96±11.85	0.058
Protein (g/day)	46.31±0.90	44.18±0.92	41.62±0.93	39.68±0.94	<0.001
All fat (g/day)	11.09±0.68	9.84±0.70	8.48±0.70	9.36±0.71	0.010
Carbohydrate (g/day)	251.0±3.38	263.0±3.45	259.14±3.50	258.74±3.53	0.271
Cholesterol (mg/day)	166.00±10.90	130.70±11.15	98.55±11.30	118.09±11.39	0.003
Calcium (mg/day)	394.85±15.90	334.28±16.26	304.05±16.48	276.57±16.61	<0.001
Phosphorus (mg/day)	789.52±15.73	734.70±16.09	689.49±16.31	620.81±16.44	<0.001
Iron (mg/day)	10.92±0.21	10.23±0.21	9.36±0.21	8.89±0.22	<0.001
Potassium (mg/day)	2,171.03±56.50	1,963.05±57.80	1,748.11±58.59	1,699.63±59.05	<0.001
Sodium (mg/day)	2,577.86±151.90	2,558.43±155.40	2,339.01±157.51	2,403.15±158.75	0.274
Zinc (mg/day)	7.58±0.14	7.50±0.15	7.21±0.15	7.11±0.15	0.008
Vitamin A (μg RE/day)	544.73±23.26	425.94±23.79	337.54±24.12	384.87±24.30	<0.001
Retinol (µg/day)	55.90±5.26	45.17±5.38	39.17±5.45	40.95±5.50	0.027
β-carotene (µg/day)	2,944.96±136.65	2,314.89±139.79	1,812.15±141.69	2,081.51±142.81	<0.001
Vitamin B_1_ (mg/day)	0.89±0.02	0.88±0.02	0.83±0.02	0.82±0.02	<0.001
Vitamin B_2_ (mg/day)	0.75±0.02	0.65±0.02	0.57±0.02	0.58±0.03	<0.001
Niacin (mg/day)	10.30±0.25	9.76±0.25	9.11±0.25	9.47±0.26	0.015
Vitamin B_6_ (mg/day)	1.17±0.03	1.11±0.03	1.02±0.03	1.00±0.03	<0.001
Folate (µg/day)	430.77±11.86	394.89±12.14	360.82±12.30	343.72±12.40	<0.001
Vitamin C (mg/day)	93.94±3.70	73.44±3.78	54.68±3.83	67.72±3.86	<0.001
Vitamin E (mg/day)	7.26±0.20	6.35±0.21	5.52±0.21	5.99±0.21	<0.001
Fiber (g/day)	16.54±0.46	15.24±0.47	13.85±0.48	12.17±0.48	<0.001

Values are presented as least squares mean±standard deviation. FBDI, food-based index of dietary inflammatory potential; RE, retinol equivalent.

1 By a general linear model after adjusting for gender, age, education, exercise, and alcohol drinking.

**Table 4.** Average of MMSE-KC scores by FBDI

MMSE-KC score		FBDI				p for trend^1^
		Quartile 1 (n=199)	Quartile 2 (n=200)	Quartile 3 (n=200)	Quartile 4 (n=199)	
Total	Model 1^2^	25.21±0.41	25.72±0.42	24.91±0.43	24.51±0.42	0.013
	Model 2^3^	25.30±0.43	25.79±0.43	25.12±0.44	24.79±0.45	0.071
Men	Model 1^4^	25.89±0.52	25.77±0.53	25.10±0.54	24.68±0.47	0.013
	Model 2^5^	25.51±0.54	25.58±0.53	25.11±0.56	24.90±0.50	0.203
Women	Model 1^4^	25.05±0.73	26.08±0.78	25.21±0.78	24.71±0.81	0.192
	Model 2^5^	25.50±0.81	26.40±0.85	25.63±0.85	24.91±0.88	0.126

Values are presented as least squares mean±standard deviation. MMSE-KC, Korean version of the Mini-Mental State Examination; FBDI, food-based index of dietary inflammatory potential.

1 By general linear model.

2 Adjusted for gender, age, and education.

3 Adjusted for gender, age, education, exercise, alcohol drinking, and use of supplements.

4 Adjusted for age and education.

5 Adjusted for age, education, exercise, alcohol drinking, and use of supplements.

**Table 5.** Odds ratios (ORs) and 95% confidence intervals (CIs) for the risk of mild cognitive impairment by FBDI

MMSE-KC group		FBDI				p for trend^1^
		Quartile 1 (n=199)	Quartile 2 (n=200)	Quartile 3 (n=200)	Quartile 4 (n=199)	
Total	Model 1^2^	1.00 (reference)	0.80 (0.50, 1.27)	1.33 (0.85, 2.08)	1.60 (1.01, 2.52)	0.012
	Model 2^3^	1.00 (reference)	0.78 (0.47, 1.29)	1.25 (0.77, 2.05)	1.50 (0.90, 2.52)	0.039
Men	Model 1^4^	1.00 (reference)	1.16 (0.53, 2.50)	1.80 (0.86, 3.79)	1.97 (1.00, 3.88)	0.059
	Model 2^5^	1.00 (reference)	0.92 (0.41, 2.08)	1.25 (0.55, 2.86)	1.20 (0.56, 2.57)	0.581
Women	Model 1^4^	1.00 (reference)	0.61 (0.34, 1.10)	1.07 (0.60, 1.90)	1.53 (0.80, 2.92)	0.047
	Model 2^5^	1.00 (reference)	0.65 (0.33, 1.26)	1.20 (0.63, 2.28)	2.22 (1.04, 4.74)	0.007

MMSE-KC, Korean version of the Mini-Mental State Examination; FBDI, food-based index of dietary inflammatory potential.

1 OR and 95% CI were obtained using multiple logistic regression analysis.

2 Adjusted for gender, age, and education.

3 Adjusted for gender, age, education, exercise, alcohol drinking, and use of supplements.

4 Adjusted for age and education.

5 Adjusted for age, education, exercise, alcohol drinking, and use of supplements.
